# Extracellular nanovesicles‐transmitted circular RNA has_circ_0000190 suppresses osteosarcoma progression

**DOI:** 10.1111/jcmm.14877

**Published:** 2020-01-10

**Authors:** Shenglong Li, Yi Pei, Wei Wang, Fei Liu, Ke Zheng, Xiaojing Zhang

**Affiliations:** ^1^ Department of Bone and Soft Tissue Tumor Surgery Cancer Hospital of China Medical University Liaoning Cancer Hospital & Institute Shenyang Liaoning Province China

**Keywords:** biomarker, *circ‐0000190*, extracellular nanovesicles, miRNA, osteosarcoma

## Abstract

Under the microenvironment, tumour progression is substantially affected by cell‐cell communication. In spite of the mediating effect of extracellular nanovesicles (EVs) on cell‐cell communication by packaging into circRNAs, the effect of EVs circRNA hsa_circ_0000190 (circ‐0000190) in osteosarcoma is still not clear. Circ‐0000190 expressions in tissues and EVs from plasma were compared between osteosarcoma patients and controls. Thereafter, receiver operating characteristic (ROC) curve was drawn and area under the curve was calculated to examine whether the diagnostic results were accurate, and the effect of EVs circ‐0000190 was dug out via the determination of cell phenotypes and animal assays. Results showed circ‐0000190 exhibited an obvious reduction in EVs and tissues of osteosarcoma patients (*P* < .05). It was also discovered that EVs encapsulated the majority of circ‐0000190, and EVs‐encapsulated circ‐0000190 could be applied to make a distinction between osteosarcoma patients and controls. Besides, EVs circ‐0000190 in osteosarcoma cells transported from normal cells weakened the capacities of osteosarcoma cells to migrate, proliferate and invade, so as to block their biological malignant behaviours (*P* < .05). In addition, under the action of EVs circ‐0000190, tumour growth was impeded and the expression of TET1 was inhibited via the competitive binding to miR‐767‐5p. In all, EVs circ‐0000190 has a good prospect as it can be regarded as a new biomarker for detecting osteosarcoma. EVs circ‐0000190 transported from normal cells to osteosarcoma cells impeded the in vitro and in vivo development of osteosarcoma, implying that EVs circ‐0000190 exerts an effect on communication between normal cells and osteosarcoma cells in the carcinogenesis process of osteosarcoma.

## 1 INTRODUCTION

Osteosarcoma is the bone tumour that most commonly affects children, adolescents and young adults,^1^ whose primary foci are the body parts such as the metaphysis of long bones of limbs where bones grow and repair actively.^2^ Previously, surgical amputation is the major treatment method for osteosarcoma, potentially triggering high morbidity rate, trauma and short long‐term survival. In the past ten years, the prognosis of patients was enhanced based on the breakthroughs in osteosarcoma, thus substantially increasing the five‐year survival rate of osteosarcoma patients to about 60%‐70%.^3^ In the past decades, however, little effect has been produced in clinical results and prognosis in spite of prominent progresses in neo‐adjuvant chemotherapy and surgery.^4^ The exact mechanisms where osteosarcoma develops and progresses are unclear none the less, so determining the molecular development mechanism of osteosarcoma becomes the top priority for diagnosing and treating the disease.^5^

Circular RNAs (circRNAs), generated from end‐to‐end ligation of RNA transcripts in the process of transcription, are closed‐loop RNAs.^6^ Despite over 40 years of investigation on circRNAs,^7^ little attention has been paid to them until recently. Enriched in a variety of tissues^8^ with the same sequence as corresponding linear isomers, circRNAs are formed *via* varying splicing mechanisms.^9^ Research has manifested that circRNAs are found in cancer cells and show abnormal expressions in such epithelial cancers as lung cancer,^10^ gastric cancer^11^ and osteosarcoma.^12^ CircRNAs consist of a ring structure that is hard to decompose compared to mRNAs,^13^ and they significantly modulate cancers, especially the function of miRNAs by combining with them.^14^ Chen S et al pointed out that *circ‐0000190* is clearly expressed in gastric cancer.^15^ Meanwhile, Feng Y et al pointed out that *circ‐0000190* regulates the miR‐767‐5p/MAPK4 pathway so as to impede multiple myeloma to progress.^16^ However, the relationship between *circ‐0000190* and osteosarcoma is still unknown.

Most types of cells can secret extracellular nanovesicles (EVs), and these EVs can also be seen in the body fluids. EVs consist of a lipid bilayer where genomic DNAs, RNAs (including mRNAs, miRNAs and other small RNAs), soluble and membrane‐bound proteins, lipids and metabolites derived from the parent cells exist.^17^ Carrying a variety of cargoes, EVs are considered to be the basic transmitters of cellular information and involved in the regulation of pleiotropic and biological functions in multicellular organisms.^18^ Hence, EVs exert a good effect as disease biomarkers and have attracted much attention in recent years.^19^ CircRNAs, small ncRNA family members, are enriched in EVs. Further, a mass of evidence shows that EVs are capable of participating in the mechanism of tumorigenesis by transmitting circRNA.^20^

In this research, the expression of *circ‐0000190* in osteosarcoma cell lines and normal osteoblast cell line was examined *via* qRT‐PCR, whose results elucidated that *circ‐0000190* was remarkably attenuated in osteosarcoma cell lines. Subsequently, the further measurement revealed that the expression *circ‐0000190* showed an obvious reduction in tissues and plasma EVs. The biological effects of EVs *circ‐0000190* on osteosarcoma have not been illustrated in reports yet. This study aims to find a potential biomarker for diagnosing osteosarcoma and to figure out whether EVs *circ‐0000190* is involved in extracellular communication so as to stimulate osteosarcoma to progress.

## 2 MATERIALS AND METHODS

### 2.1 Study design and subjects

Plasma samples were obtained from 60 osteosarcoma subjects and 60 healthy controls, and the corresponding 60 pairs of paracancerous normal tissues and tumour tissues were collected from osteosarcoma subjects in Liaoning Cancer Hospital & Institute, followed by analysis. This research received the informed consent from all the subjects and gained the approval of the institutional review board of Liaoning Cancer Hospital & Institute.

### 2.2 Cell lines

Human‐derived osteoblasts hFOB1.19 and osteosarcoma cell lines (SAOS‐2, MG63, U2OS, SJSA1 and HOS) were provided by Cell Bank, Chinese Academy of Science, Shanghai. Cells were maintained in DMEM (Gibco BRL, Grand Island, NY, USA) with 10% FBS (Gibco BRL) at 37°C with 5% CO_2_.

### 2.3 Cell transfection

With reference to the instructions, cells were subjected to transfection by the transfection reagent Lipofectamine 2000 provided by Invitrogen, Carlsbad, CA, USA *circ‐0000190* overexpression plasmid, vector NC and miR‐767‐5p mimics were synthesized by GeneChem (Shanghai, China). The lentiviral vectors with *circ‐0000190*/NC were synthesized by GeneChem.

### 2.4 Cell proliferation assay

Approximately 4.0 × 10^3^ MG63 and U2OS cells undergoing transfection with *circ‐0000190* overexpression/NC vectors or incubated with EVs (from 1.5 × 10^6^ hFOB1.19 cells) were plated in 96‐well plates. The Cell Counting Kit‐8 (Dojindo Laboratories, Kumamoto, Japan) was utilized to determine cell proliferation based on manufacturer's protocol. The absorbance at 450 nm was measured using the Infinite M200 spectrophotometer (Tecan, Switzerland).

Cells seeded into 96‐well plates with 5 × 10^3^ cells/well were labelled with 50 μmol/L medium labelled with 5‐ethynyl‐2'‐deoxyuridine (EdU; RiboBio, Guangzhou, China). Two hours later, cells were subjected to 4% paraformaldehyde and 0.5% Triton X‐100 and incubated with anti‐EdU working solution. Nuclei were dyed with DAPI. Five randomly selected views in each well were captured using a fluorescent microscope for calculating EdU‐positive cells. We performed all experiments in triplicate.

### 2.5 Migration‐related assays

Based on the methods mentioned above, Transwell invasion and migration assays were carried out for analysis.^21^

### 2.6 RNase R digestion

After kept at 37°C for 15 minutes, 3 units of RNase R (Epicentre Biotechnologies) were added to 1 μg RNA, respectively, to degrade the linear RNA. Following RNase R treatment, qRT‐PCR was performed to detect the expressions of GAPDH and *circ‐0000190*.

### 2.7 Dual‐luciferase reporter assay

After seeded into the 24‐well‐plate, luciferase reporters (10 ng) and miR‐767‐5p mimics (80 nmol/L) or mimics NC were then applied to transfect cells (1 × 10^4^) using Lipofectamine 2000 (Invitrogen, Shanghai, China), followed by the luciferase reporter assay with the Dual‐Luciferase Reporter Assay System (Promega, Shanghai, China).

### 2.8 RNA binding protein immunoprecipitation assay

RNA binding protein immunoprecipitation (RIP) assay was strictly carried out with reference to the instructions of the Millipore kit (Millipore, Bedford, MA, USA). Following cell lysis, each reaction system was added with 8 μg detection antibodies, in which cells were incubated at 4°C overnight, followed by reheating to room temperature for 1 hour. Then, the complex was captured using Protein G magnetic beads, and the buffer was washed to extract RNA. The extracted RNA was then reversely transcribed, and qRT‐PCR was adopted for RNA level detection.

### 2.9 Separation of EVs

The hFOB1.19, MG63 and U2OS cells were plated onto 10‐cm dishes at a concentration of 1.5 × 10^6^ cells/ dish. After 48 hours, the culture media were discarded, and the cells were washed three times in phosphate‐buffered saline (PBS). Next, the cells were cultured in serum‐free media. After 72 hours, cell culture media were collected and 10 ml plasma from each sample was also collected for separation of EVs. The plasma and culture medium were collected and centrifuged at 3000 *g* for 15 minutes to remove cells and cellular debris. Then, we filtered the supernatant through a 0.22‐μm PVDF filter (Millipore). In the case of filtered plasma, an appropriate volume of Thrombin was added to the plasma and centrifuged at 100,000×*g*, 5 minutes to make them compatible with ExoQuick exosome precipitation. Then, we added the appropriate volume of ExoQuick exosome precipitation solution (System Biosciences) to the Thrombin‐treated plasma and filtered culture medium. After refrigeration for 24 hours, the ExoQuick/biofluid mixture was centrifuged at 1500 *g* for 30 minutes, and the supernatant was removed. The EVs appear as a beige or white pellet at the bottom of the vessel.

### 2.10 TEM

Prior to TEM analysis, 100 μl of PBS was added for EVs suspension and 5% glutaraldehyde for incubation at the incubation temperature and kept at 4°C. After that, EVs samples were placed dropwise on a copper grid coated with carbon, followed by 30 seconds of soaking in 2% phosphotungstic acid solution (pH 7.0) in accordance with the preparation procedures of TEM samples. Under a transmission electron microscope (TecnaiG2 Spirit Bio‐Twin; FEI, USA), the preparations were observed.

### 2.11 EVs labelling

Extracellular nanovesicles from 1.5 × 10^6^ cells were suspended in 100 μl of PBS with 1 ml of mixed PKH67, a green fluorescent marker (Sigma, in Diluent C), followed by incubation at room temperature for 4 minutes. Subsequently, EVs labelling was terminated through the addition of 2 ml of BSA (0.5%), and Exosome Spin Columns (MW 3000; Thermo Fisher Scientific, Shanghai, China) was used to remove unincorporated dye from EVs labelling reactions. The ExoQuick Exosome Precipitation Solution was added for separation of stained EVs. As the negative control, EVs were collected without PKH67 dye. Thereafter, 9.6 ml of basal medium was taken for EVs suspension, and 250 μl was placed onto the subconfluent layer of MG63 and U2OS cells. Then, the cells were incubated for 3 hours at 37°C, rinsed and fixed at room temperature. DAPI (Sigma) was added for 10 minutes of nucleus staining, and a fluorescence microscope (Zeiss, LSM700B, Germany) was utilized to observe the stained cells.

### 2.12 RNA isolation and qRT‐PCR

First of all, TRIzol reagent (Invitrogen, CA, USA) was utilized for separating the total RNAs from tissues and cell lines and the exoRNeasy Midi Kit (Qiagen, Valencia, CA, USA) for extracting EVs RNA from plasma and culture medium based on the manufacturer's scheme. Then cDNA synthesis was achieved by means of a high‐capacity cDNA reverse transcription kit (Thermo Fisher Scientific, Vilnius, Lithuania), followed by qRT‐PCR through an ABI 7900 system (Applied Biosystems, CA, USA) and SYBR Green assays (TaKaRa Biotechnology, Dalian, China). Then, normalization of the expression level of circRNA was achieved with GAPDH as a reference control. 2^−ΔCt^ method was adopted to measure the fold change in circRNA expression. Primer sequences are displayed below: *circ‐0000190*: F 5'‐GAGGGCAGCTGAAGTCACAC‐3', R 5'‐ACCAGTGCAATGACATGAGC‐3', GAPDH: F 5'‐CCGGGAAACTGTGGCGTGATGG‐3', R 5'‐AGGTGGAGGAGTGGGTGTCGCTGTT‐3', *TET1*: F 5'‐CATCAGTCAAGACTTTAAGCCCT‐3', R 5'‐CGGGTGGTTTAGGTTCTGTTT‐3'.

### 2.13 Animal assays

*circ‐0000190*/NC lentiviral vectors were transfected into MG63 cell lines (1 × 10^7^ cells in 0.1 ml of PBS) stably, and the right flank of 5‐week‐old male nude mice received subcutaneous injection of these cells. Then tumour growth was detected every other day, and the tumour size and weight were examined following execution of mice 15 days later. To investigate the effect of EVs*circ‐0000190* in vivo, the male nude mice were only subcutaneously injected with MG63 cells in the right flank, and when the volume of tumours reached 100 mm^3^, the centre of tumours was injected with 10 μg EVs from hFOB1.19 cells that were transfected with *circ‐0000190*/NC vectors every other day. Fifteen days later, tumours in different groups were examined after all mice were killed. Animal assays were approved by the Institutional Animal Care and Use Committee of China Medical University.

### 2.14 IHC

Representative areas were selected *via* H&E staining, and anti‐*TET1* (Abcam, Shanghai, China) or anti‐*Twist* was used for IHC according to manufacturer's programme.

### 2.15 Western blot analysis

Cells or EVs were lysed in RIPA buffer (CWBIO, Beijing, China) with protease and phosphatase inhibitors (CWBIO). Identical quantities of proteins were electrophoresed by SDS‐PAGE, transferred onto PVDF membranes and incubated with primary antibodies specific for *TSG101* (Abcam, Shanghai, China), *CD63* (Abcam), *TET1* (Abcam), *Twist* (Abcam) and *GAPDH* (Abcam) at 4°C overnight, followed by incubation with appropriate HRP‐conjugated secondary antibodies at room temperature for 1 hour. Signals were detected by Immobilon ECL substrate (Millipore, Germany), and the images were acquired using an Optimax X‐ray Film Processor (Protec, Germany).

### 2.16 Statistical analysis

Quantitative data are presented as the mean ± SEM. Statistical comparisons were performed by the chi‐squared test and Fisher's exact test when testing small samples using SPSS version 20.0 software (SPSS, Chicago, IL, USA). The Pearson test was performed to assess correlations between *circ‐0000190* expression and *TET1*. Student's *t* test was used for difference analysis between the two groups of experiments in vivo and in vitro. The receiver operating characteristic (ROC) curve reflected the area under the curve values for EVs *circ‐0000190* in plasma. The optimal cut‐off value of *circ‐0000190* was determined by the maximal Youden index. For all tests, a *P*‐value of less than .05 was considered as statistically significant.

## 3 RESULTS

### 3.1 Characteristics of patients

Characteristics of the osteosarcoma patients and healthy controls are displayed in Table 1. Differences in age, gender and smoking status were not discovered between patients and controls (*P* > .05).

**Table 1:** The characteristics of the osteosarcoma cases and healthy controls

Variables	Case (n = 60)	Control (n = 60)	*P*‐value
Age (years) (mean ± SD)			
<50	45	41	.4178
≥50	15	19	
Gender			
Male	38	40	.7019
Female	22	20	
Smoking status			
Never	50	48	.6370
Ever	10	12	

Note

Two‐sided chi‐squared for all variables between osteosarcoma cases and controls.

### 3.2 Characteristics of *circ‐0000190* in osteosarcoma

**Figure 1:**
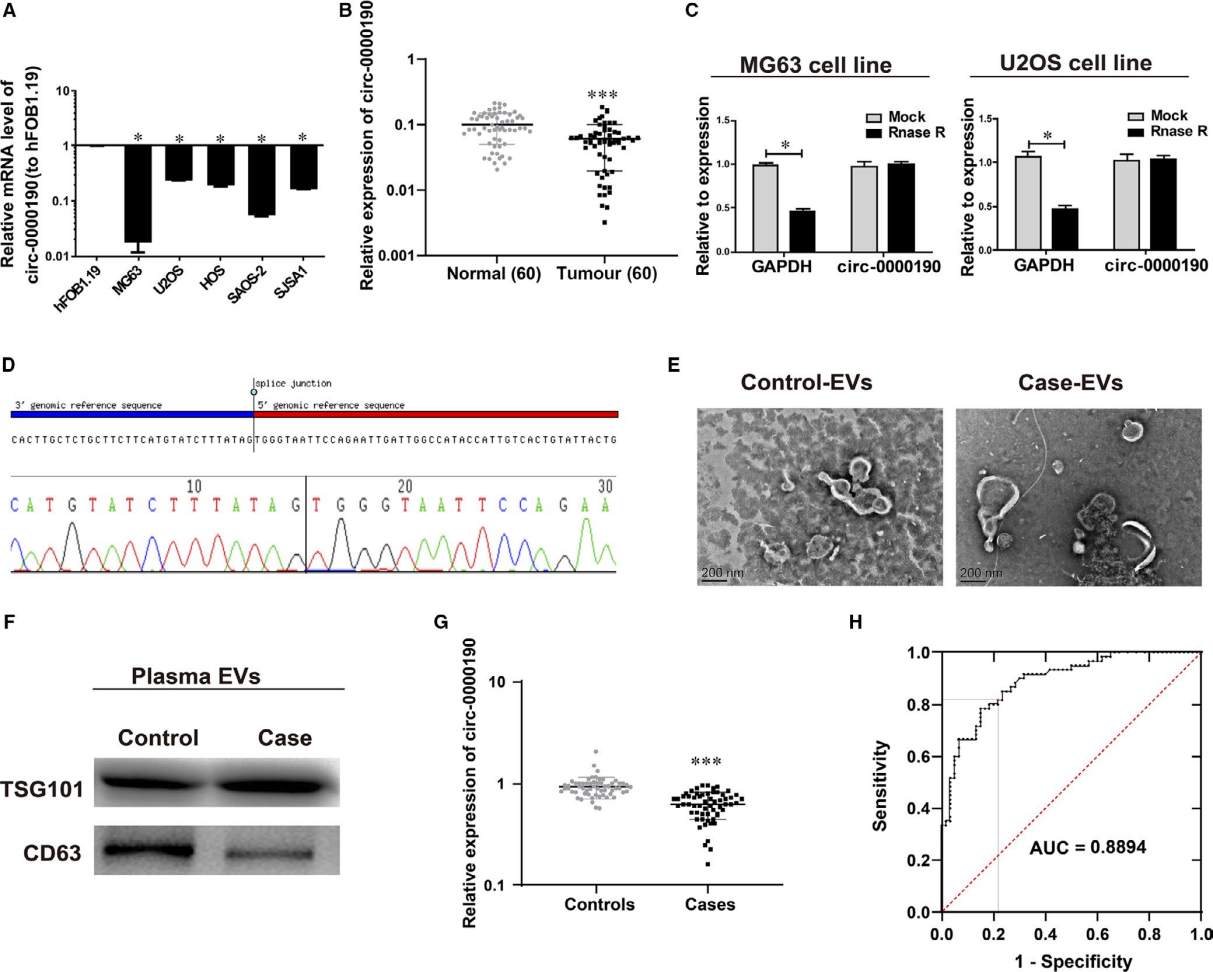
Characteristics of *circ‐0000190* in osteosarcoma. Extracellular nanovesicles are separated from the plasma of cases and controls. A, The mRNA level of *circ‐0000190* in osteosarcoma cell lines and normal osteoblasts (hFOB1.19). B, qRT‐PCR detection of *circ‐0000190* in osteosarcoma tumour tissues and paired adjacent normal tissues. C, CircRNA has obvious resistance to RNase R digestion in osteosarcoma cell line. D, The sequence of *circ‐0000190* in circBase is the same as that shown in Sanger sequencing. E, EVs from the plasma of cases and controls displayed in micrographs. F, *TSG101* and *CD63* in circulating EVs detected *via* Western blotting. G, *circ‐0000190* in plasma EVs examined through qRT‐PCR. H, EVs *circ‐0000190* signature analysed by ROC curves. *P < .05, ***P < .001

The expression of *circ‐0000190* in osteosarcoma cell line and human‐derived osteoblasts hFOB1.19 was measured *via* qRT‐PCR, and the results showed that *circ‐0000190* expression was significantly reduced in osteosarcoma cell line (Figure 1A). Subsequently, we detected the content of *circ‐0000190* in osteosarcoma tissue and adjuvant normal tissue, the results of which manifested that *circ‐0000190* expression was evidently reduced in osteosarcoma tumour tissue (Figure 1B). Meanwhile, further analysis by Pearson chi‐squared test or Fisher's exact test indicated lower expression of *circ‐0000190* was correlated with bigger tumour size (≥5 cm), advanced staging (IIB/III) and distant metastasis (Table 2). The circular nature of *circ‐0000190* was confirmed by treatment of total RNAs with RNase R. This assay suggested that *circ‐0000190* is indeed a circRNA which is resistant to RNase R digestion (Figure 1C). We confirmed that the *circ‐0000190* sequence amplified by the primer was identical to its sequence in circbase through Sanger sequencing (Figure 1D).

**Table 2:** Association of *circ‐0000190* expression with clinicopathological features of osteosarcoma

Feathers	Number	Low	High	*P*‐value
All cases	60	30	30	
Age (years)				
<18	35	18	17	1.0000
≥18	25	12	13	
Gender				
Male	28	15	13	.7961
Female	32	15	17	
Tumour size (cm)				
<5	23	7	16	.0326^*^
≥5	37	23	14	
Histological subtype				
Osteoblastic	6	3	3	.8609
Chondroblastic	12	7	5	
Fibroblastic	25	11	14	
Mixed	17	9	8	
Distant metastasis				
Absent	28	8	20	.0019^*^
Present	32	22	10	
Anatomic location				
Tibia/femur	34	16	18	.6023
Elsewhere	26	14	12	
Clinical stage				
I‐IIA	33	12	21	.0195^*^
IIB‐III	27	18	9	

Note

Total data from 60 tumour tissues of osteosarcoma patients were analysed. For the expression of *circ‐0000190* was assayed by qRT‐PCR, the median expression level was used as the cut‐off. Data were analysed by chi‐squared test and Fisher's exact test. *P*‐value in bold indicates statistically significant.

Then, EVs RNA was extracted from plasma, and EVs from plasma of cases and controls were separated and their characteristics were analysed. According to TEM results, cases had the same size of EVs with controls (50‐150 nm, Figure 1E). The existence of exosome markers, TSG101 and CD63 was proved by Western blotting (Figure 1F). Results mentioned above indicated that EVs *circ‐0000190* might act as a useful biomarker for discriminating patients with osteosarcoma from healthy controls. Thereafter, the expression of EVs *circ‐0000190* in plasma was detected, and it was discovered that cases had a lower expression of EVs *circ‐0000190* than controls (*P* < .05, Figure 1G). Additionally, the corresponding ROC curves were used to investigate the underlying value of EVs *circ‐0000190* as a non‐invasive biomarker. The effect of EVs *circ‐0000190* on diagnosing restenosis is presented in Figure 1H. The under area of ROC curve was 0.889 (95% CI: 0.833‐0.946). The Youden index was 0.769, and the sensitivity and specificity were 85.0% and 78.3%, respectively.

### 3.3 Effect of *circ‐0000190* on osteosarcoma cellular phenotype

**Figure 2:**
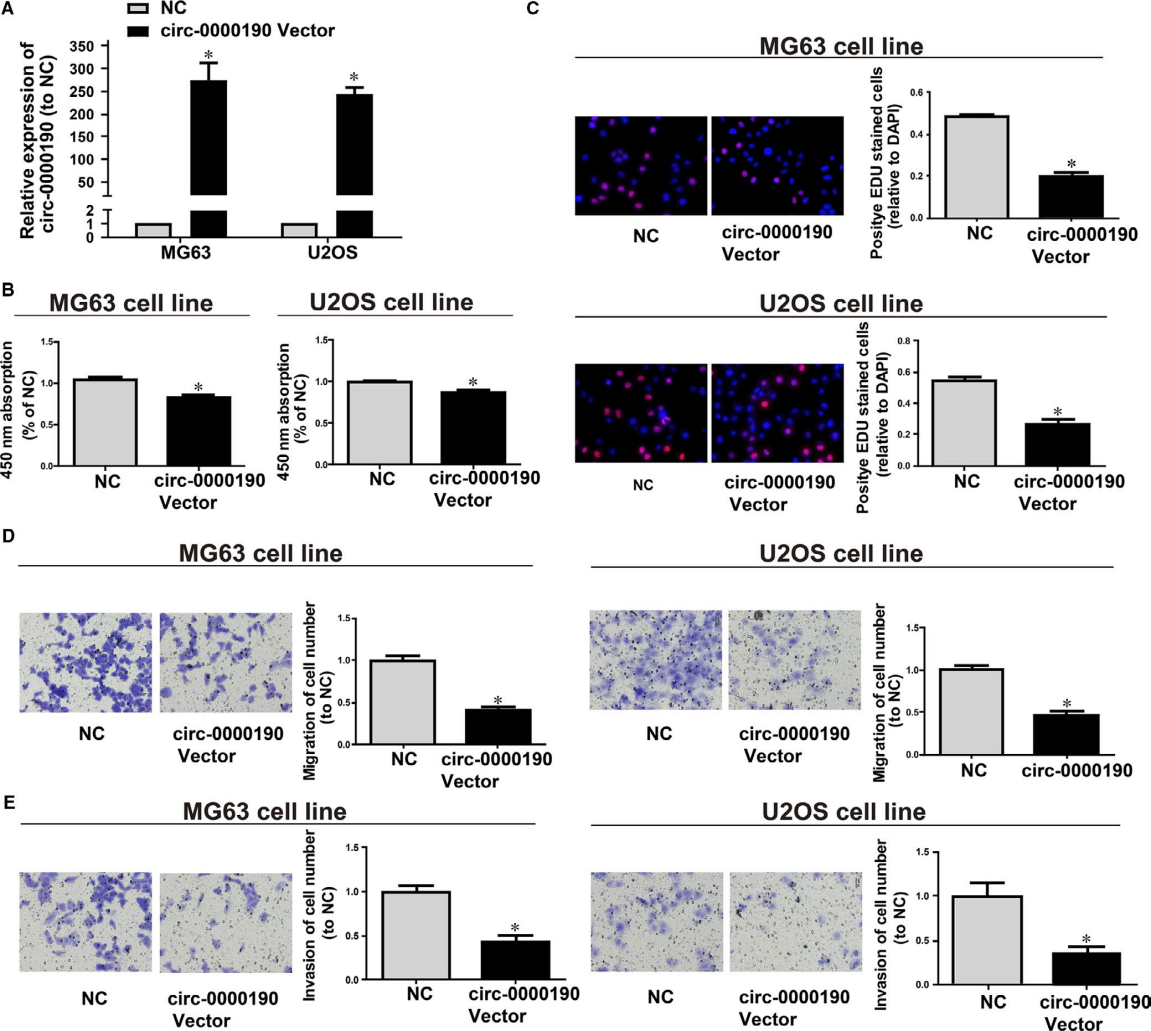
Effect of *circ‐0000190* on osteosarcoma cellular phenotype. *circ‐0000190* plasmids or NC vectors are used to transfect MG63 and Hep3b cells. A, qRT‐PCR measurement of the *circ‐0000190* mRNA level. B, CCK8 assay determination of cell viability. C, EdU assay detection of cell proliferation. D, Transwell assay detection of cell migration. E, Cell invasion detection of cell invasion. *P < .05. NC, negative control vector. Each experiment was carried out three times

The biological effect of *circ‐0000190* in vitro was explored in this study since *circ‐0000190* was found to be lowly expressed in tissues and EVs in plasma from osteosarcoma cases. After that, MG63 and U2OS cells were transfected with *circ‐0000190* overexpression plasmids or negative control vectors (NC), and qRT‐PCR was carried out to determine *circ‐0000190* expression (Figure 2A). In the first place, 24 hours of *circ‐0000190* overexpression prominently impeded the proliferation of MG63 and U2OS cells in comparison with those in cells transfected with NC vectors (Figure 2B, 2). Moreover, both Transwell migration assay and cell invasion assay indicated the inhibiting effect of *circ‐0000190* on osteosarcoma cell migration and invasion (Figure 2D, 2).

### 3.4 EVs *circ‐0000190* mediates intercellular communication

**Figure 3:**
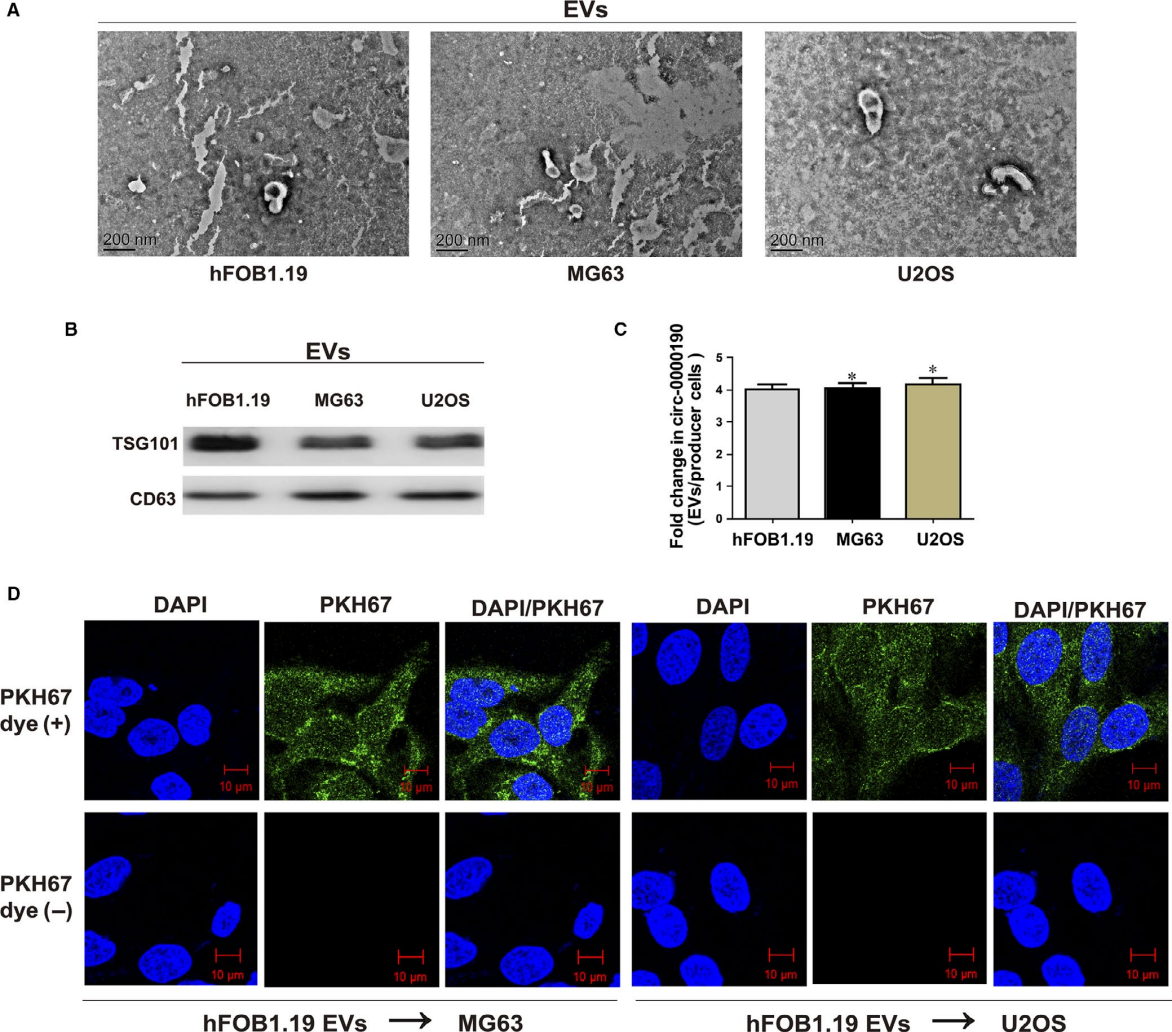
Extracellular nanovesicles *circ‐0000190* mediates intercellular communication. Extracellular nanovesicles (EVs) are separated from the medium of hFOB1.19, U2OS and MG63 cells. A, EVs from hFOB1.19 (left), MG63 (middle) and U2OS cells (right, bars = 200 nm) shown in micrographs. B, TSG101 and CD63 in EVs of cell lines detected *via* Western blotting. C, Fold change in *circ‐0000190* between EVs of hFOB1.19, U2OS and MG63 and their producer cells measured through qRT‐PCR. D, PKH67‐labelled or non‐labelled EVs derived from hFOB1.19 cells; green represents PKH67, and blue represents nuclear DNA after DAPI staining. U2OS and MG63 cells are subjected to 3 h of incubation with EVs from hFOB1.19 cells. Results are indicated as mean ± SD. *P < .05. Each experiment was carried out three times

The existing pattern of extracellular *circ‐0000190* was investigated, and TEM was carried out to determine the size of EVs (Figure 3A). According to Western blotting results, TSG101 and CD63 existed (Figure 3B), and it was demonstrated that *circ‐0000190* expression was significantly higher in hFOB1.19 cells than in U2OS and MG63 cells (Figure S1A). Meanwhile, EVs *circ‐0000190* expression exhibited a notably lower expression in U2OS and MG63 cells than in hFOB1.19 cells (Figure S1B). Furthermore, *circ‐0000190* levels displayed approximately fourfold increases in EVs in comparison with producer cells (Figure 3C). Hence, EVs from U2OS and MG63 cells possessed less *circ‐0000190* than those from hFOB1.19 cells, which was identical to the findings in this study, and *circ‐0000190* overexpression was detected in healthy controls. Then, PKH67 was used to label EVs from hFOB1.19 cells, and the labelled EVs were applied to incubate recipient cells (U2OS and MG63 cells) for 3 hours. PKH67 was found to be located in the cytoplasm of recipient cells (Figure 3D).

### 3.5 Effect of EVs *circ‐0000190* on osteosarcoma cellular phenotype

**Figure 4:**
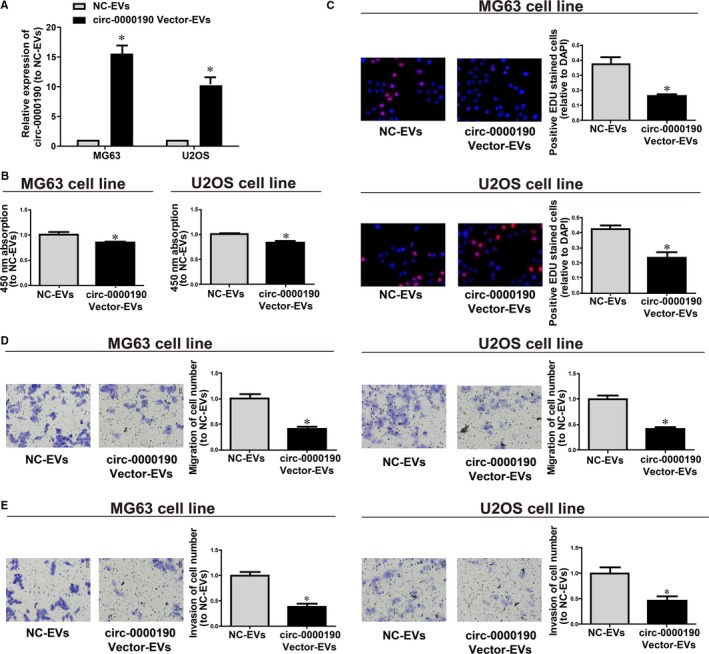
Effect of extracellular nanovesicles *circ‐0000190* on osteosarcoma cellular phenotype. Extracellular nanovesicles (EVs) from hFOB1.19 cells transfected with *circ‐0000190* overexpression plasmids or NC vectors, namely *circ‐0000190*‐EVs and NC‐EVs, respectively. Their EVs were extracted and added to the MG63 and U2OS cells for 24 h. A, qRT‐PCR determination of the *circ‐0000190* mRNA level. B, CCK8 assay detection of cell viability. C, EdU assay detection of cell proliferation. D, Transwell assay detection of cell migration. E, Cell invasion detection of cell invasion. Results are indicated as mean ± SD. *P < .05. Each experiment was carried out three times

*circ‐0000190* silencing frequently occurred in cases and their cell lines rather than in controls, indicating that *circ‐0000190* is potentially be a tumour suppressor. As proved above, the transmission of *circ‐0000190* from hFOB1.19 cells into MG63 and U2OS cells was achieved through EVs, and it was then predicted that the biological functions of MG63 and U2OS cells could be changed by EVs *circ‐0000190* from hFOB1.19 cells. In order to figure out the functions of EVs *circ‐0000190*, EVs were separated from hFOB1.19 cells transfected with *circ‐0000190* overexpression plasmids or NC vectors, namely *circ‐0000190*‐EVs or NC‐EVs, which were then added at 100 μg/ml to MG63 and U2OS cells for 24 hours. The levels of *circ‐0000190* in MG63 and U2OS cells exposed to *circ‐0000190*‐EVs showed an evidently increase compared with those exposed to NC‐EVs for 24 hours (Figure 4A). Based on the results, *circ‐0000190*‐EVs were capable of remarkably impede the proliferation of MG63 and U2OS cells (Figure 4B, 4). Additionally, both Transwell migration assay and invasion assay indicated the inhibiting effect of *circ‐0000190*‐EVs on osteosarcoma cell migration and invasion (Figure 4D, 4).

### 3.6 Subcellular distribution of *circ‐0000190*

**Figure 5:**
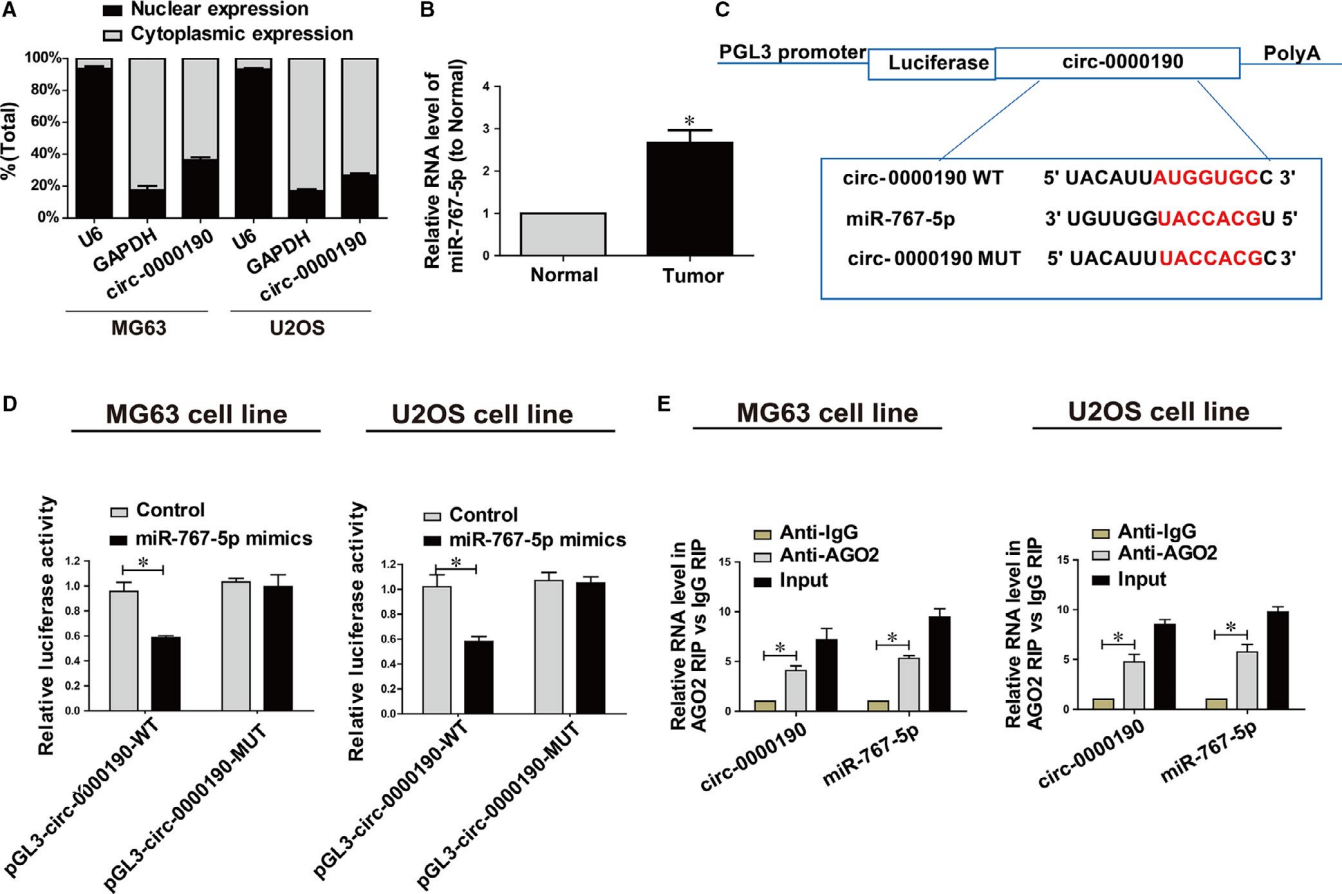
*circ‐0000190* directly interacts with miR‐767‐5p. A, Cytoplasmic and nuclear levels of *circ‐0000190* in U2OS and MG63 cells analysed by qRT‐PCR. B, MiR‐767‐5p expression in osteosarcoma tissues and adjacent normal tissues detected *via* qRT‐PCR. C, Bioinformatics evidence of binding of miR‐767‐5p onto 3'‐UTR of *circ‐0000190*. D, Dual‐luciferase reporter gene assay in MG63 and U2OS cells after transfection with negative control or miR‐767‐5p mimics, renilla luciferase vector pRL‐SV40 and the reporter constructs. E, RIP experiments for the amount of *circ‐0000190* and miR‐767‐5p in MG63 and U2OS cells. Data are indicated as mean ± SD. *P < .05

Subcellular distribution of circRNA determines its biological function. To confirm the cellular localization of *circ‐0000190*, we isolated osteosarcoma cells into cytoplasmic and nuclear fractions, with GAPDH and U6 as controls, respectively. QRT‐PCR results showed that 63.7% and 73.5% of *circ‐0000190* were distributed in the cytoplasmic fraction of MG63 and U2OS cells, respectively (Figure 5A). We may conclude that *circ‐0000190* participated in the development of osteosarcoma through post‐transcriptional regulation.

### 3.7 *circ‐0000190* is targeted by miR‐767‐5p

Given that *circ‐0000190* was primarily located in the cytoplasmic fraction, we hypothesized that *circ‐0000190* may act as a ceRNA in the development of osteosarcoma. QRT‐PCR data revealed that miR‐767‐5p expression was higher in osteosarcoma tumour tissues, which was contrary to the expression trend of *circ‐0000190* (Figure 5B). Through bioinformatics prediction (RegRNA, Starbase), we found that sequences in miR‐767‐5p that were highly matched to *circ‐0000190* 3'UTR. Based on these binding sequences, pGL3‐*circ‐0000190*‐WT and pGL3‐*circ‐0000190*‐MUT were established (Figure 5C). Luciferase activity was obviously down‐regulated in MG63 and U2OS cells cotransfected with *circ‐0000190* WT and miR‐767‐5p mimics, while it did not change after transfection with *circ‐0000190* MUT (Figure 5D). RIP analysis was carried out to elucidate whether *circ‐0000190* was involved in RNA‐containing ribonucleoprotein complex. QRT‐PCR results showed that *circ‐0000190* was enriched in anti‐Ago2 antibody than controls. Similar results were yielded in miR‐767‐5p (Figure 5E). It is suggested that miR‐767‐5p can bind to *circ‐0000190* in vitro.

### 3.8 *circ‐0000190* regulates *TET1*, the target gene of miR‐767‐5p

**Figure 6:**
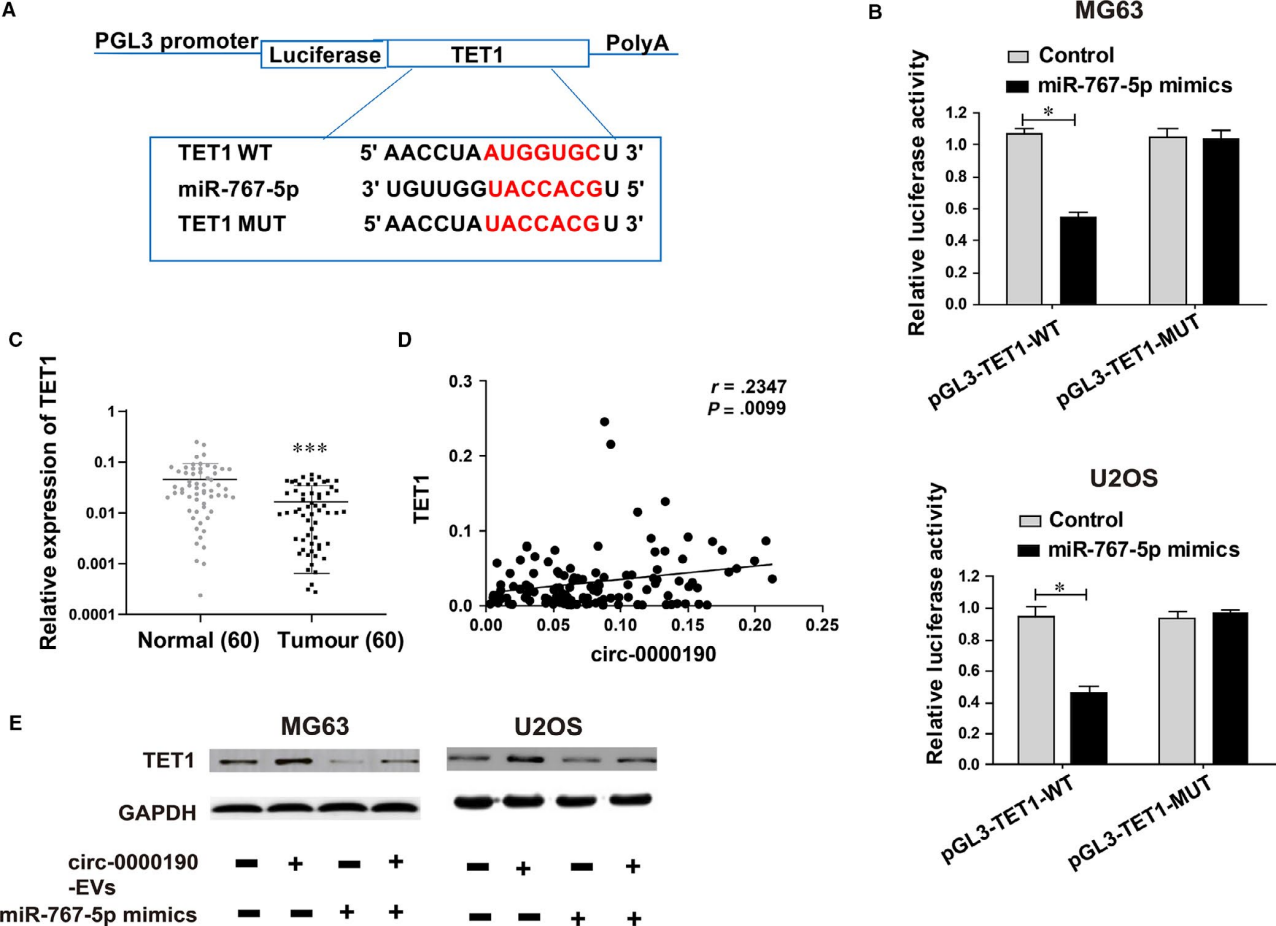
*TET1* is the direct target of miR‐767‐5p. A, The putative miRNA binding sites in the *TET1* sequence. B, Dual‐luciferase reporter gene assay is carried out to verify the direct target sites. C, *TET1* expression in osteosarcoma tissues and adjacent normal tissues detected by qRT‐PCR. D, Bivariate correlation analysis of the relationship between *circ‐0000190* and *TET1* expression level. E, *TET1* in MG63 and U2OS cells with *circ‐0000190*‐EVs and/or miR‐767‐5p mimics examined *via* Western blotting. Data are indicated as mean ± SD. *P < .05, ***P < .001

The potential role of miR‐767‐5p in the development of osteosarcoma was explored by the screening of miR‐767‐5p target genes using bioinformatics prediction (TargetScan, Starbase, RegRNA). Finally, *TET1* was selected for further analyses. After construction of luciferase plasmids pGL3‐*TET1*‐WT and pGL3‐*TET1*‐MUT, they were cotransfected with miR‐767‐5p mimics or NC in MG63 and U2OS cells, respectively (Figure 6A). Luciferase activity of the WT reporter was inhibited, but that of MUT reporter did not change (Figure 6B). The above findings imply that *TET1* is a potential target gene of miR‐767‐5p. Subsequently, *TET1* expression in osteosarcoma cell line was determined *via* qRT‐PCR. The mRNA levels of *TET1* were remarkably inhibited in osteosarcoma tumour tissues (Figure 6C). Interestingly, there was a significant correlation of the expression levels between *circ‐0000190* and *TET1* (Figure 6D). Besides, miR‐767‐5p mimics lowered *TET1* expression, while EVs *circ‐0000190* prominently reversed this effect (Figure 6E).

### 3.9 Effects of *circ‐0000190* overexpression and EVs *circ‐0000190* on tumour in vivo

**Figure 7:**
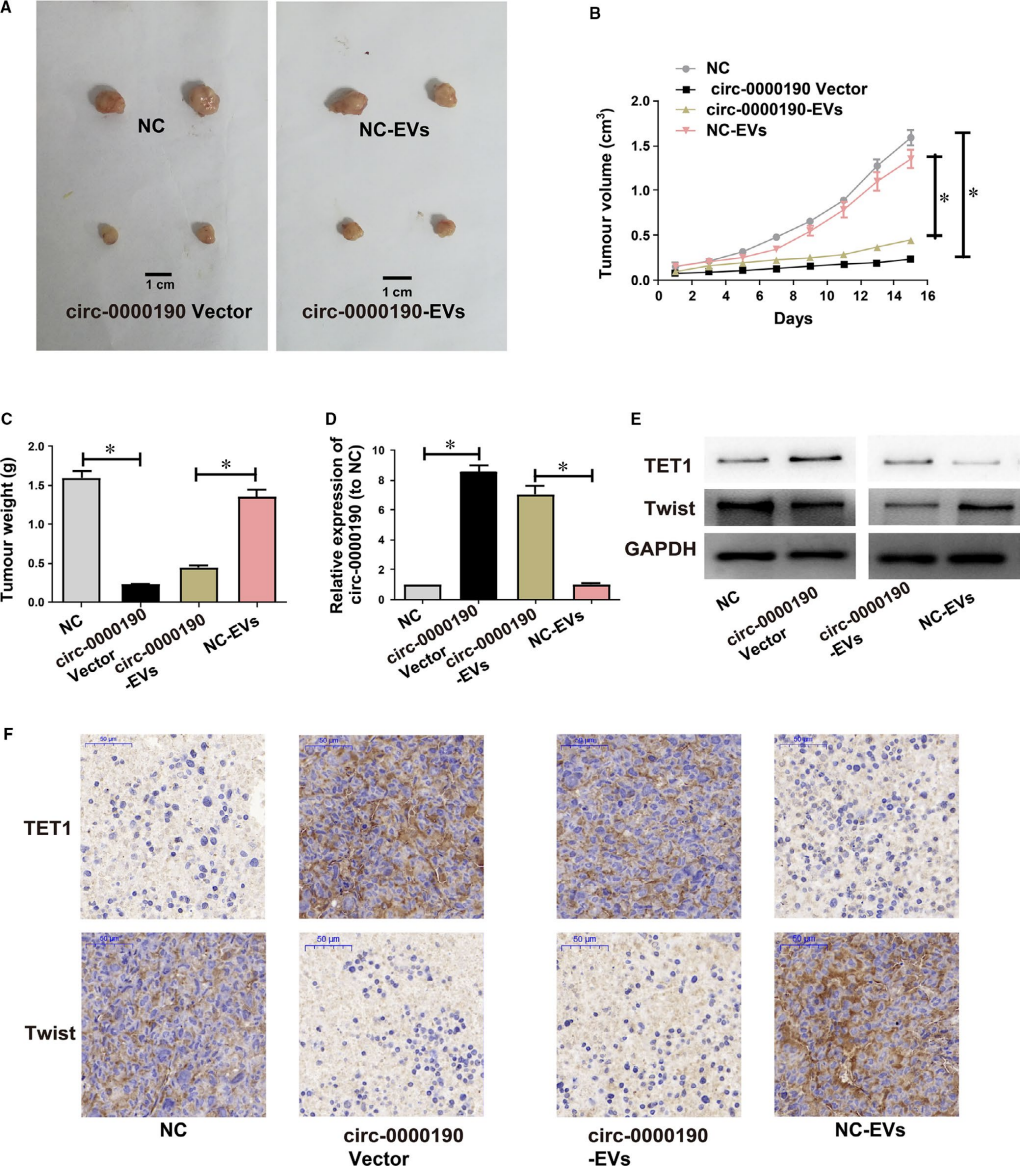
Effects of *circ‐0000190* overexpression and extracellular nanovesicles *circ‐0000190* on tumour in vivo. *circ‐0000190*/NC lentiviral vectors are used to transfect MG63 cells, namely *circ‐0000190* vectors and NC, respectively. Extracellular nanovesicles (EVs) are separated from hFOB1.19 cells transfected with *circ‐0000190*/NC lentiviral vectors, namely *circ‐0000190*‐EVs and NC‐EVs, respectively. A, The xenografts from nude mice injected with NC, *circ‐0000190*‐EVs, *circ‐0000190* vector and NC‐EVs. B, The tumour volumes are detected every other day after injection. C, 15 days later, the tumour weights in nude mice are measured. D, *circ‐0000190* expressions in tumour tissues of nude mice treated with NC, *circ‐0000190* vectors, *circ‐0000190*‐EVs and NC‐EVs detected through qRT‐PCR. E, *TET1* and *Twist* in tumour tissues detected *via* Western blotting. F, *TET1* and *Twist* expressions in tumour tissues examined *via* IHC. Results are indicated as mean ± SD. *P < .05

The effects of *circ‐0000190* overexpression and EVs *circ‐0000190* on osteosarcoma in vivo were further investigated through the injection of *circ‐0000190*/NC‐EVs from hFOB1.19 cells transfected with *circ‐0000190*/NC lentiviral vectors and from MG63 cells transfected with *circ‐0000190*/NC lentiviral vectors into nude mice. Based on the analysis in vitro, the mean tumour weight and average tumour volume were markedly decreased in *circ‐0000190* overexpression group (Figure 7A‐C) in comparison with those in NC group. The results also denoted that the tumour tissues of nude mice injected with *circ‐0000190*‐EVs also decreased the tumour size and weight (Figure 7A‐C). Besides, *circ‐0000190* expression displayed an increase in the tumour tissues of mice injected with *circ‐0000190* vectors and *circ‐0000190*‐EVs (Figure 7D). Moreover, Western blotting detection and IHC for *TET1* were carried out to detect *TET1* expression, the results of which illustrated that *TET1* expression was evidently stimulated in the models of *circ‐0000190* overexpression and *circ‐0000190*‐EVs (Figure 7E, 7). In the meantime, the EMT marker gene *Twist* was significantly inhibited in *circ‐0000190* overexpression and *circ‐0000190*‐EVs models (Figure 7E, 7), implying that perhaps *TET1* plays a role in the pathogenesis of osteosarcoma through the modulation on the EMT process.

## 4 DISCUSSION

By analysing osteosarcoma microarray data,^22^ we found that *circ‐0000190* is down‐regulated in osteosarcoma cell lines. We then verified the results of the microarray by cell experiments. Through detecting the human tissue plasma EVs *circ‐0000190*, *circ‐0000190* was discovered to be decreased in osteosarcoma tissues and plasma. In addition, normal cells were shown in this study to be able to encapsulate *circ‐0000190* into EVs and secrete into osteosarcoma cells, significantly inhibiting the vicious behaviour of osteosarcoma cells by reducing the proliferation, invasion and migration of cancer cells. Besides, EVs *circ‐0000190* blocked tumour growth in vivo. Therefore, EVs *circ‐0000190* can be a potential biomaker for osteosarcoma detection.

Existing evidence has proved that the physiological condition of the donor cells can be reflected by EVs circRNAs, and these EVs circRNAs will trigger a sequence of cellular responses after they were captured by recipient cells.^23^ Furthermore, it has been reported that EVs circRNAs are potential cancer biomarkers for different cancer patients^24^ which indicates that EVs circRNAs can be applied for cancer identification as potential biomarkers.

In this research, the size and shape of plasma EVs were analysed *via* TEM, and TSG101 and CD63 (exosome markers) were used to verify exosomes.^25^ After the measurement of the expression level of *circ‐0000190* in plasma EVs, it was interestingly found that the level of EVs *circ‐0000190* was decreased in osteosarcoma patients. In spite of the function of EVs *circ‐0000190* in detection osteosarcoma confirmed by NC results, large‐sample research is still needed to ensure the diagnostic accuracy. Accumulating evidence has shown that various biochemical cellular processes including proliferation and migration are under the control of circRNAs.^26^ Based on the obtained results, the putative tumour suppressor function of *circ‐0000190* in human osteosarcoma cells was examined. Numerous assays we conducted revealed that overexpression of *circ‐0000190* weakened cell proliferation, migration and invasion. In the meantime, animal assays also evidenced the function of *circ‐0000190* to inhibit the tumour in osteosarcoma.

Data in this study verified that *circ‐0000190* was generated mainly through secretion from cells via EVs. Cancer‐related reports involve a large quantity of data on the effects of EVs circRNAs.^27^ EVs significantly influence cell‐cell communication and jointly change the physiological function of the recipient cells with bioactive factors, including circRNAs.^28^ For instance, EVs from normal cells transferred *PTENP1* to bladder cells, which suppresses bladder cancer progression.^29^ With efforts made in this study, it was concluded that *circ‐0000190* level was elevated in EVs from normal cells, about four times greater than that in producer cells. It was revealed in fluorescence microscopy that PKH67‐labelled EVs from normal cells could transfer into osteosarcoma cells. The results mentioned above imply that EVs *circ‐0000190* secreted by hFOB1.19 cells probably transferred to the surrounding osteosarcoma cells. Despite wide investigation on EVs circRNAs transported from cancer cells to normal cells, reports on EVs circRNAs transferred from normal cells into cancer cells are rare. The results of this study demonstrated that *circ‐0000190*‐EVs from hFOB1.19 cells elevated *circ‐0000190* expression, blocked the proliferation of osteosarcoma cells, and inhibited tumour growth in vivo, which are identical to the results in previous research. In a word, these study results indicate that *circ‐0000190* is transported from normal cells to osteosarcoma cells via EVs in a direct way and has a regulatory role in the biological functions of osteosarcoma in vitro and in vivo.

QRT‐PCR results showed that *circ‐0000190* was primarily distributed in the cytoplasmic fraction of osteosarcoma cells. We may conclude that *circ‐0000190* participated in the development of osteosarcoma through post‐transcriptional regulation. Hence, it was speculated that *circ‐0000190* may be a ceRNA, participating in tumorigenesis of osteosarcoma. *TET1* belongs to the TET (ten‐eleven translocation) family. *TET1* plays a crucial role in the DNA methylation process and gene activation.^30^ Research of Duan H et al have shown that *TET1* inhibits EMT of osteosarcoma cells through activating Wnt/β‐catenin signalling inhibitors DKK1 and SFRP2.^31^ Our findings revealed that EVs *circ‐0000190* has the ability to modulate *TET1* levels by competitively binding to miR‐767‐5p.

In summary, the study results denote that EVs *circ‐0000190* is a new biomarker that is potentially valuable for diagnosing osteosarcoma. Additionally, normal cells released EVs containing *circ‐0000190*, and EVs *circ‐0000190* was transmitted from normal cells to osteosarcoma cells, and exogenous *circ‐0000190* relieved the malignant phenotype of osteosarcoma cells both in vitro and in vivo. In addition, EVs *circ‐0000190* may induce miR‐767‐5p to modulate *TET1* and impede osteosarcoma progression. Above all, this study reveals that EVs *circ‐0000190* mediates extracellular communication during carcinogenesis of osteosarcoma.

## CONFLICTS OF INTEREST

All of the contributors in this study declared no conflict of interest.

## AUTHOR CONTRIBUTIONS

Xiaojing Zhang designed the experiments; Yi Pei performed the experiments; Shenglong Li and Fei Liu wrote the paper; all authors discussed the results and contributed to the modification of the manuscript.

## Data Availability

The data in the current study are available from the corresponding authors on reasonable request.
