# Supplementary material for: Extracellular nanovesicles‐transmitted circular RNA has_circ_0000190 suppresses osteosarcoma progression
Source: J Cell Mol Med. 2020 Jan 10;24(3):2202–14. doi: 10.1111/jcmm.14877 (PMC7011131; doi:10.1111/jcmm.14877)
Supplement: Supplementary file 1 [file JCMM-24-2202-s001.docx]

**Supplementary Figure 1**

**
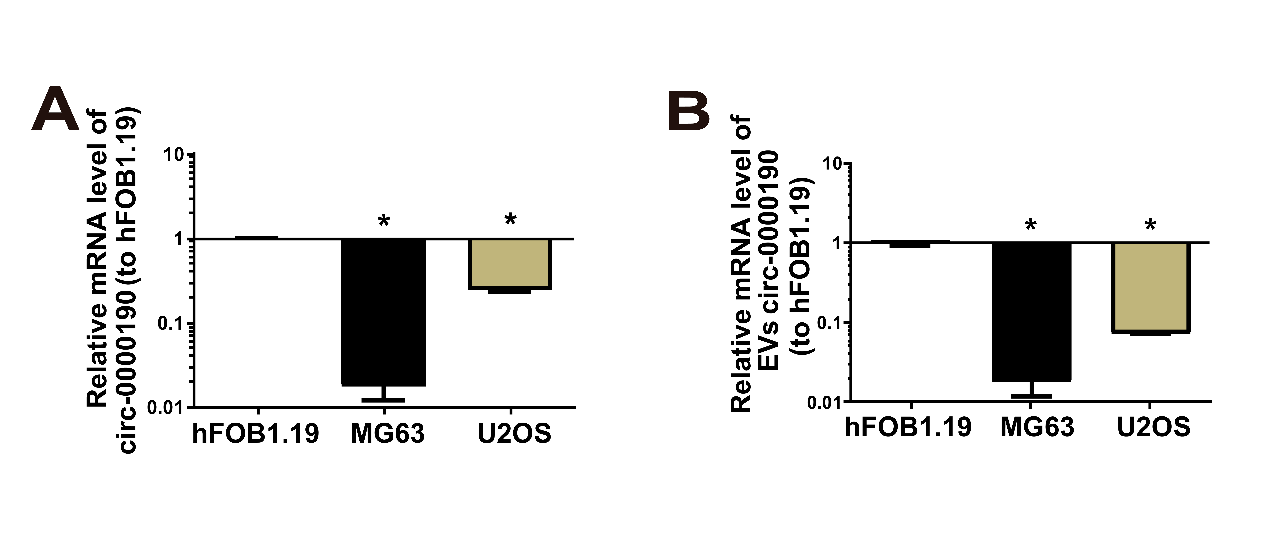
**

**Supplementary Figure 1. Relative mRNA level of hsa_circ_0000190.** (A) qRT-PCR detection of hsa_circ_0000190 in hFOB1.19, MG63 and U2OS cells. (B) qRT-PCR detection of EVs hsa_circ_0000190 of hFOB1.19, MG63 and U2OS. Data are the mean ± SEM. *P < 0.05.
